# A Metabolic Plasticity-Based Signature for Molecular Classification and Prognosis of Lower-Grade Glioma

**DOI:** 10.3390/brainsci12091138

**Published:** 2022-08-26

**Authors:** Ming-Chun Yang, Di Wu, Hui Sun, Lian-Kun Wang, Xiao-Feng Chen

**Affiliations:** 1 Department of Neurosurgery, 1st Affiliated Hospital, Harbin Medical University, Harbin 150001, China; 2 Department of Obstetrics and Gynecology, 1st Affiliated Hospital, Harbin Medical University, Harbin 150001, China; 3 Pharmaceutical Experiment Teaching Center, College of Pharmacy, Harbin Medical University, Harbin 150081, China; 4 Department of Neurology, Heilongjiang Province Hospital, Harbin 150001, China

**Keywords:** glioma, metabolic signature, prognosis

## Abstract

Background: Glioma is one of the major health problems worldwide. Biomarkers for predicting the prognosis of Glioma are still needed. Methods: The transcriptome data and clinic information on Glioma were obtained from the CGGA, TCGA, GDC, and GEO databases. The immune infiltration status in the clusters was compared. The genes with differential expression were identified, and a prognostic model was developed. Several assays were used to detect RPH3A’s role in Glioma cells, including CCK-8, colony formation, wound healing, and transwell migration assay. Results: Lower Grade Glioma (LGG) was divided into two clusters. The immune infiltration difference was observed between the two clusters. We screened for genes that differed between the two groups. WGCNA was used to construct a co-expressed network using the DEGs, and four co-expressed modules were identified, which are blue, green, grey, and yellow modules. High-risk patients have a lower overall survival rate than low-risk patients. In addition, the risk score is associated with histological subtypes. Finally, the role of RPH3A was detected. The overexpression of RPH3A in LGG cells can significantly inhibit cell proliferation and migration and regulate EMT-regulated proteins. Conclusion: Our study developed a metabolic-related model for the prognosis of Glioma cells. RPH3A is a potential therapeutic target for Glioma.

## 1. Introduction

Glioma is composed of aberrant growing glial cells, which is one of the leading causes of cancer deaths [1]. Lower grade Gliomas (LGG) account for about 10–20% of all primary brain tumors [2]. Despite the advances in the current surgical and medical treatments for LGG, the outcomes of patients are variable [3]. Therefore, an effective prognostic model to distinguish the heterogeneity of Glioma is urgently needed to improve clinical outcomes. 

Metabolites are closely associated with physiological and pathological changes. Tumors have specific metabolites that ensure uncontrolled growth. Some specific metabolites are altered in the processes of tumor progression. Tumor cells enhance anabolic pathways, enabling them to use carbon sources other than glucose. Through metabolic reprogramming, tumors can easily source energy through glycolysis [4]. In addition to differentiated Glioma cells, Glioma stem-like cells can switch metabolic pathways in response to metabolic stress. The metabolic heterogeneity and plasticity of Glioma stem-like cells are, therefore, characteristic [5].

The tumor immune microenvironment plays a critical role in Glioma. Glioma has an immune-suppressive nature, which is related to enhanced immunosuppressive factors, such as PD-1, PD-L1, indolamine 2,3-dioxygenase, etc. [6,7]. Many immune-based therapeutics have been developed, especially immune-checkpoint inhibitors [8]. The tumor immune microenvironment also affects the response of a tumor to cancer therapies [9]. Moreover, LGG can be classified histologically and molecularly based on the immune status. The immune status was associated with the prognosis of LGG [10]. The immune-related signatures have a prognostic value for LGG [11]. 

It is the tumor microenvironment that determines the metabolic interactions between tumor cells and the immune system, resulting in the metabolic heterogeneity of tumors and affecting the prognosis of patients [12]. Therefore, comprehensively exploring the role of metabolic heterogeneity in LGG is of great importance. In this study, differentially expressed genes between different metabolic subtypes were identified. A metabolic-related prognostic model was constructed and validated. Among the model component genes, the overexpression of RPH3A has great potential for its anti-cancer effect on Glioma. To validate our hypothesis, a cell-line-based assay was carried out to analyze the role of RPH3A in Glioma. Our research provided an effective prognostic model to facilitate a precise clinical therapy for Glioma and a potential therapeutic agent for Glioma treatment. 

## 2. Methods and Materials

### 2.1. Glioma Datasets

The mRNA microarray and mRNA sequencing data of 301 and 693 Glioma patients with corresponding clinical information were downloaded from the Chinese Glioma Genome Altas (CGGA, http://www.cgga.org.cn/download.jsp, 1 August 2021), which were marked as “CGGA array (301)” and “CGGA RNAseq (693)” in our work. From the Genomic Data Commons, we downloaded information on the gene expression and clinical characteristics of LGG patients from the Cancer Genome Altas (TCGA). (GDC, https://portal.gdc.cancer.gov/, 1 August 2021). According to the WHO 2006 classification of grade II Glioma (astrocytoma, oligoastrocytoma, or oligodendroglioma), the expression levels were detected by microarray. The Gene Expression Omnibus was used to download its classification (GEO, http://www.ncbi.nlm.nih.gov/geo, 1 August 2021) (GSE107850). 

### 2.2. Metabolic Enrichment Based on Clustering

A total of 86 metabolic pathways were downloaded from the Kyoto Encyclopedia of Genes and Genomes (KEGG, https://www.genome.jp/kegg/, 1 August 2021) database. For each sample, a single-sample Gene Set Enrichment Analysis (ssGSEA) was utilized to calculate the enrichment score of each metabolic gene set using the R package “GSVA” with default parameters based on the transcriptomic data [13]. As a result, each sample achieved 86 metabolic enrichment scores.

The patients were clustered using K-means using the metabolic pathway scores; cluster 1 had a better prognosis than cluster 2. By using principal component analysis (PCA), we explored the differences between the two clusters.

### 2.3. Comparison of Immune Infiltration between Clusters

According to CIBERSORT, the number of tumor-infiltrating immune cells was counted for each type [14]. In a mixed cell population, CIBERSORT estimates the abundance of different types of cells using gene expression data. A comparison between the CGGA and TCGA cohorts was carried out to determine the range of 22 infiltrating immune cells. The two clusters were compared according to the infiltration of immune cells using the Wilcoxon rank-sum test.

### 2.4. Analysis of Functional Differences between Clusters

The CGGA array (301) differentially expressed genes (DEGs) were identified using the “limma” package in the R software. A total of 688 genes were differentially expressed with FDR < 0.001 and |logFC| > 1.5. 

We performed pathway and process enrichment analysis for DEGs using the Metascape web-based tool (https://metascape.org/gp/index.html, 1 August 2021). To ensure its content is current, Metascape is updated monthly. Default settings were used for the Metascape analysis.

### 2.5. Identification of Meaningful Co-Expression Module

Weighted gene co-expression network analysis (WGCNA) corresponds to a data reduction method and unsupervised classification method [15,16]. With the help of the “WGCNA” package in the R software, the co-expression network was constructed based on the DEGs expression profiles. An analysis of the CGGA array (301) cohort was conducted to identify which co-expression module was most relevant to the tumor grade. An association method based on module traits was applied. Using cluster analysis, the phenotypes correlated with the gene modules.

### 2.6. Prognostic Model Established

In the CGGA array (301) cohort, we performed a univariate Cox proportional regression model to identify the genes associated with OS in the “blue” module. A total of 239 genes with *p* < 0.0001 were considered for the subsequent analysis. To identify the significant prognostic genes, we used least absolute shrinkage and selection operator (LASSO) as a variable selection procedure in a Cox regression model. The standard error (SE) was selected as one standard deviation above the minimum criteria. A multivariate Cox regression coefficient estimation based on 13 optimized genes and correlations was used to calculate a risk score formula: Risk score = (exp Gene1 × coef Gene1) + (exp Gene2 × coef Gene2) + … +(exp Gene13 × coef Gene13).

### 2.7. Survival Analysis and Correlation Analysis of Histological Subtypes and Risk Score

According to the median risk score assigned to the patients with Gliomas, the high-risk patients and the low-risk patients were classified as high-risk and low-risk patients. A log-rank test was used to assess the difference in the survival time between the patients with high and low risk. For the presentation of the results, Kaplan–Meier plots were used.

The patients were grouped according to tumor grade, Eastern Cooperative Oncology Group (ECOG) score, and histological types. A Wilcoxon rank sum test was used to determine whether there were any differences in risk scores among the groups.

### 2.8. Quantitative Real-Time PCR and Cell Culture

The Glioma cell lines (SW1783 and SW1088) were provided by BeNa Culture Collection (Shanghai, China). A high-glucose DEME (Gibco, Grand Islan, NE, USA) and L-15 medium containing 10% fetal bovine serum was used for the culture of the SW1783 (grade III astrocytoma) and SW1088(an astrocytoma cell line with a fibroblast-like morphology) cells. SW1783 and SW1088 were used for an in vitro model for LGG, similar to previous research [17,18,19]. For the plasmid construction and transfection for RPH3A overexpression, the plasmid pcDNA™3.1 (Sino biological, Shanghai, China) was designed to construct RPH3A using a Lipofectamine 2000 transfection reagent (Thermos Fisher Scientific, Waltham, MA, USA) for transfection. A TRIzol lysis method was used to take the total RNA from the cells, and a first-strand cDNA synthesizing kit from Thermos Scientific was used to reverse transcribe the RNA into cDNAs (Thermos Scientific, Waltham, MA, USA). According to the manufacturer’s protocol (SYBR Green Master Mix, Vazyme), we performed quantitative real-time PCR to detect the RPH3A mRNA levels. The 2^−ΔΔ^Ct method was used to calculate the gene expression levels. Sangon (Shanghai, China) provided the primers, and the sequences for the qPCR were as follows: for RPH3A, the forward primer was 5ʹ-GTCAAGCTCTGGCTGA-3ʹ, and the reverse primer was 5ʹ-GCAGCCTCCGATGTAA-3ʹ for β-actin, the forward primer was 5ʹ-TGACATCAAGAAGGTGG-3ʹ, and the reverse primer was 5ʹ-TTACTCCTTGGAGGCC-3ʹ.

### 2.9. Wound Healing and Transwell Migration Assay

A pipette tip (10 μL) was used to draw the surface of the cell layer in the SW1783 and SW1088 cells grown for 24 h on plates. In the next step, the cells were transfected with RPH3A-overexpression and empty vector. Images were captured at 0 and 72 h using a microscope (Phenix, Nanjing, China). After measuring the distance of the injury area after 72 h, a relative migration rate was calculated by normalizing the distance at 0 h. 

We performed a transwell migration assay (Labselect, Guangzhou, Guangdong, China) using SW1783 and SW1088 mixed with a serum-free media and injected into the upper layer of the chamber. The complete medium filled the transwell migration assay chamber. Following 24 h of culture under suitable conditions, the transwell migration assay chambers were fixed and stained with 4% paraformaldehyde and 0.1% crystal violet (Aladdin, Shanghai, China). Counting was conducted on the cells at the bottom of the chambers.

### 2.10. Western Blotting

To extract the proteins from SW1783 and SW1088, a BCA Protein assay kit (Beyotime, Shanghai, China) was used to determine the protein concentration. The separation of the proteins was performed with an electrophoresis gel comprising sodium dodecyl sulfate and polyacrylamide (12.5%). In order to block the polyvinylidene fluoride membrane containing the proteins, 5% nonfat milk was applied to the membrane for two hours. The membrane was incubated overnight at 4 °C with specific primary antibodies (Vimentin, N-cadherin, and β-actin; Zenbio, Chengdu, Sichuan, China). After incubation with the secondary antibodies, the protein blots were detected with an ECL Western Blotting Substrate (Solarbio, Shanghai, China).

### 2.11. Cell Colony Formation Assay

By performing colony formation experiments, it was determined whether or not the cells could grow independently. A total of 1000 cells, transfected with either RPH3A or the empty vector, were plated into 6-well plates. After 10–14 days, 4% paraformaldehyde was used to fix the colonies. After staining with 0.01% crystal violet, the colonies were counted to determine whether the cells had started to grow.

### 2.12. Cell Proliferation Assay

A proliferation assay was performed on the SW1783 and SW1088 cells transfected with RPH3A or an empty vector using the Cell Counting Kit-8 (CCK-8). A 96-well plate was seeded with SW1783 and SW1088 (2000 cells/well) 24 h following transfection. They were incubated at 37 °C for two hours, followed by a measurement of the absorbance at 450 nm with 10 μL of the CCK-8 regent. Repeated assays were carried out every 24 h.

## 3. Results

### 3.1. Stratification of Glioma Based on Metabolic Pathway 

**Figure 1:**
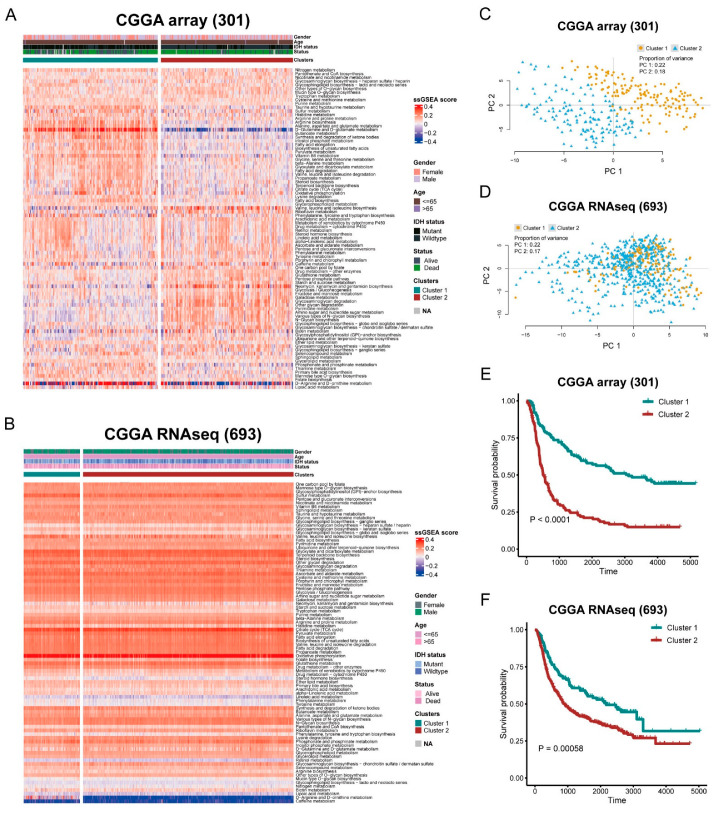
Stratification of Glioma patients with distinct metabolic activity. (**A**,**B**) Heatmap shows normalized ssGSEA enrichment score and individual characters of Glioma patients in CGGA array (301) and CGGA RNAseq (693) cohorts between two clusters. (**C**,**D**) PCA of two clusters for the enrichment score of metabolic pathways in CGGA array (301) and CGGA RNAseq (693) cohorts. (**E**,**F**) Kaplan-Meier curves of OS between cluster 1 and cluster 2 in CGGA array (301) and CGGA RNAseq (693) cohorts. CGGA array (301): HR = 2.783693; CGGA RNAseq (693): HR = 1.576667.

To understand the metabolic heterogeneity of Glioma, we first assessed the metabolic dysregulation in the Glioma samples. ssGSEA was performed based on the metabolic transcriptional profiles, and a quantitative evaluation for metabolic dysregulation was formed. According to the unsupervised K-means clustering, we identified two heterogeneous subtypes in the CGGA cohorts (cluster 1 and cluster 2) (Figure 1A,B) and the TCGA-LGG cohort (Figure S1A). PCA revealed that the patients had a distinctive metabolic pathway enrichment score between the two clusters (Figure 1C,D and Figure S1B). Next, we explored the difference in prognosis, and survival analysis showed that cluster 1 had a significantly better overall survival (OS) than cluster 2 in the CGGA array (301) and the CGGA RNAseq (693) cohorts (Figure 1E,F, *p* < 0.0001, *p* = 0.00058), the same phenomenon appearing in the TCGA-LGG cohort (Figure S1C, *p* < 0.05). 

### 3.2. Differential Metabolism and Immune Infiltration between Clusters

**Figure 2:**
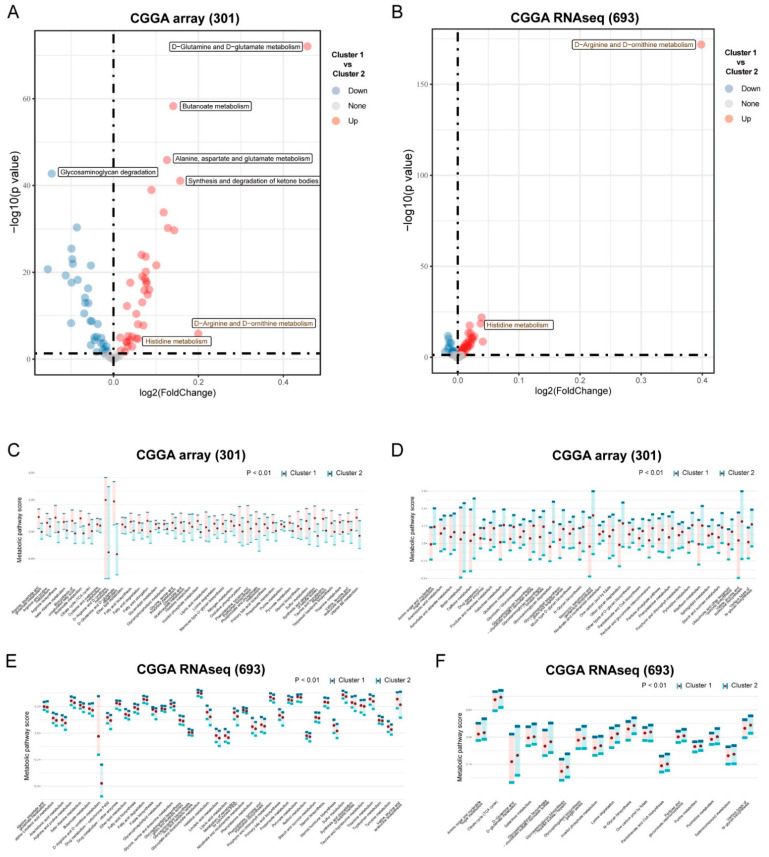
Differentially active metabolic pathway between subtypes. (**A**,**B**) Volcano plot for differentially active metabolic pathway between cluster 1 and cluster 2 in CGGA array (301) and CGGA RNAseq (693) cohorts according to fold-change method. Red points mean the pathways that upregulated in cluster 1 compared with cluster 2, and blue points mean downregulation. Number of differential up- and down-regulated pathways in (**A**) is 38 and 32, respectively; number of differentials up- and down-regulated pathways in (**B**) is 36 and 16, respectively. (**C**–**F**) Distribution of pathway enrichment score between two clusters in CGGA array (301) and CGGA RNAseq (693) cohorts, one-sided Wilcoxon rank-sum test was used to evaluate the difference.

To compare the metabolite pattern between the subtypes, we identified differential active pathways (Figure 2A,B and Figure S1D). “D−Arginine and D−ornithine metabolism” and “Histidine metabolism” were significantly upregulated in cluster 1, both in the CGGA array (301) and the CGGA RNAseq (693) cohorts (*p* < 0.00001). In addition, many metabolic pathways were significantly upregulated in cluster 1 according to the CGGA array (301) cohort, such as “D−Glutamine and D−glutamate metabolism”, “Butanoate metabolism”, “Alanine, aspartate and glutamate metabolism”, and “Synthesis and degradation of ketone bodies”, and the “Glycosaminoglycan degradation” pathway was significantly downregulated in cluster 1. A pathway enrichment score of 39 metabolic pathways in cluster 1 was significantly higher than that of cluster 2 in the CGGA array (301) cohort (Figure 2C, *p* < 0.01), and a total of 33 metabolic pathways were lower in cluster 1 (Figure 2D, *p* < 0.01). A pathway enrichment score of 40 metabolic pathways in cluster 1 was significantly higher than that of cluster 2 in the CGGA RNAseq (693) cohort (Figure 2E, *p* < 0.01), and a total of 17 metabolic pathways were lower in cluster 1 (Figure 2F, *p* < 0.01). The pathway enrichment score of eight metabolic pathways in cluster 1 was significantly higher than that of cluster 2 in the TCGA-LGG cohort (Figure S2A, *p* < 0.01), and 70 metabolic pathways were lower in cluster 1 (Figure S2B, *p* < 0.01).

**Figure 3:**
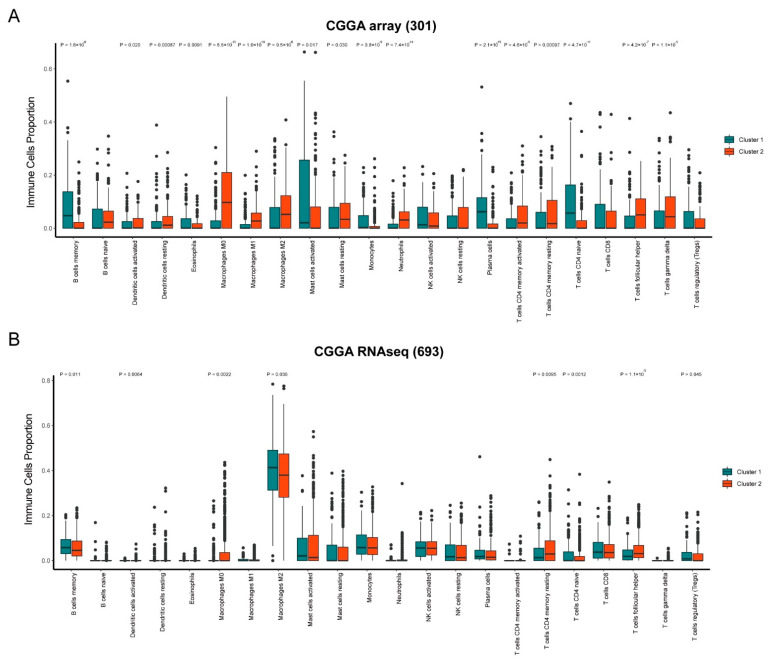
Divergence of immune microenvironment between subtypes. Distribution of 22 immune cells infiltration between cluster 1 and cluster 2 in (**A**) CGGA array (301) and (**B**) CGGA RNAseq (693) cohorts.

Next, we investigated the divergence in the immune microenvironment between the two clusters. CIBERSORT was used to quantify the abundance of 22 immune cells from the Glioma samples. Overall, 77.27% (17/22) of the immune cells showed a significant difference in infiltration between the two clusters in the CGGA array (301) cohort (Figure 3A, *p* < 0.05). The infiltration of “T cells CD4 memory activated” (*p* = 4.6 × 10^−5^), “T cells CD4 memory resting” (*p* = 0.00097), and “T cells follicular helper” (*p* = 4.2 × 10^−7^) in cluster 1 were significantly less than cluster 2 in the CGGA array (301) cohort, as well as “Macrophages M0” (*p* = 6.5 × 10^−15^), “Macrophages M1” (*p* = 1.0 × 10^−10^), and “Macrophages M2” (*p* = 9.5 × 10^−6^). The infiltration of “B cells memory” in cluster 1 was significantly more than in cluster 2. A total of eight immune cells significantly infiltrated the two clusters in the CGGA RNAseq (693) cohort (Figure 3B, *p* < 0.05). Similar to the CGGA array (301), the infiltration of “T cells CD4 memory resting” (*p* = 0.0095), “T cells follicular helper” (*p* = 1.1 × 10^−5^), and “Macrophages M0” (*p* = 0.0022) in cluster 1 were significantly less than cluster 2 in the CGGA RNAseq (693) cohort, and “B cells memory” (*p* = 0.011) in cluster 1 was significantly more. A total of eight immune cells showed significantly different infiltration between the two clusters in the TCGA-LGG cohort (Figure S2C).

### 3.3. Mining of Meaningful Module 

**Figure 4:**
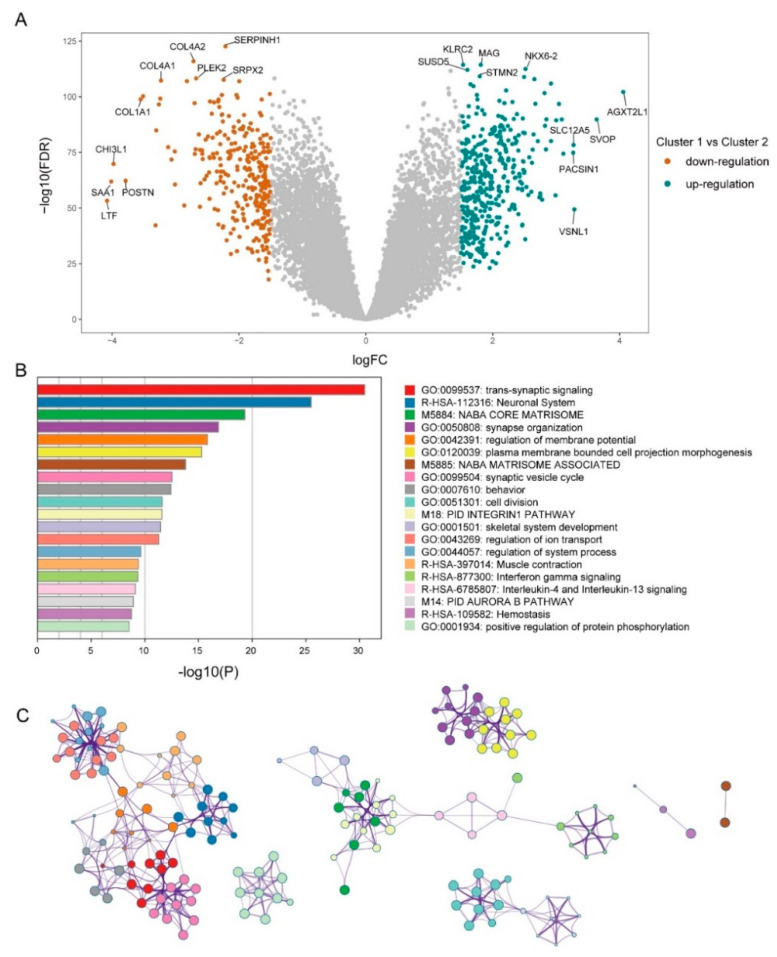
Functional analysis for DEGs. (**A**,**B**) Heatmap shows DEGs between two clusters. Red points mean upregulated in cluster 1, turquoise points mean downregulation. (**B**) An analysis of pathway and process enrichment was conducted using the following ontologies: KEGG Pathway, GO Biological Processes, Reactome Gene Sets, Canonical Pathways, CORUM, TRRUST, DisGeNET, PaGenBase, Transcription Factor Targets, and COVID-19. Based on the graphical representation, the top 20 enrichments with *p* < 0.01 were displayed. (**C**) Edges connect terms that have a similarity > 0.3.

To identify the genes that play critical roles in mediating metabolic subtype differentiation, we performed differential expression analysis between the two clusters. The volcano plot showed 688 DEGs in cluster 1 compared with cluster 2 (Figure 4A). Our study utilized Metascape to identify the pathways and processes that are enriched by DEGs to identify the functional processes regulated by DEGs comprehensively. They were significantly enriched in many crucial biological events, such as “trans-synaptic signaling”, “Neuronal System”, “NABA CORE MATRISOME”, “synapse organization”, and the “regulation of membrane potential” (Figure 4B). Some of the significantly enriched pathways were closely interlinked (Figure 4C).

**Figure 5:**
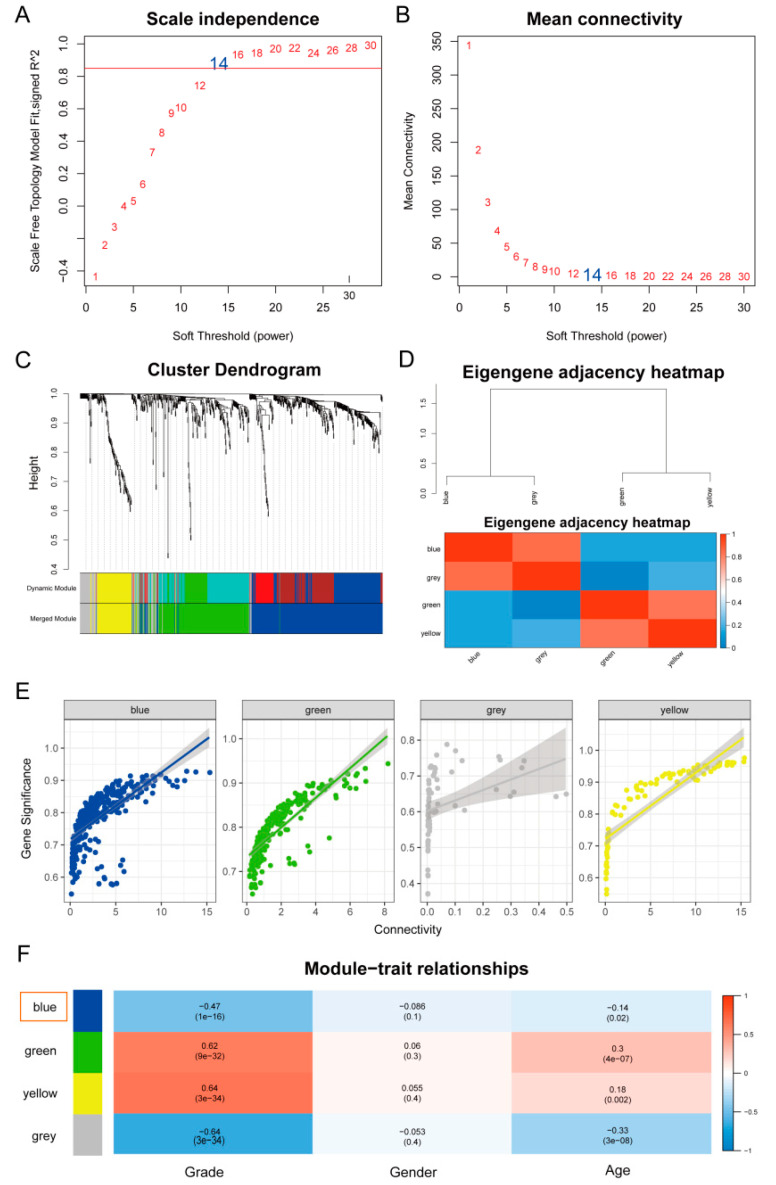
Differentially active metabolic pathway between subtypes. (**A**) Scale-free fit index versus soft-thresholding power. (**B**) Mean connectivity versus soft-thresholding power. (**C**) An adjacency-based clustering of dissimilarity-based clustering of genes is presented as a tree (dendrogram). According to the dynamic tree cut method, the colored row below the dendrogram indicates module membership, together with the color assigned to the merged modules and the original colors. (**D**) WGCNA module correlations, colored red for positive correlations and blue for negative correlations. (**E**) The connectivity in each module. (**F**) The rows correspond to Modu Eigengenes and the columns to clinical phenotypes. Each cell contains the corresponding correlation and *p* values. Color legends correspond to correlations in Table S1.

To identify the genes that were significantly associated with the clinical factors, we performed WGCNA to construct a co-expressed network according to the expression of DEGs in the CGGA array (301) cohort. To ensure average connectivity and independence, we screened the power values between 1 and 30 for each module. A scale-free network was ensured by setting the power to 14 once the scale-free R2 reached 0.9 at this time in the study (Figure 5A,B). Four co-expressed modules were identified, and the number of genes in each module was as follows: 318 in blue, 221 in green, 65 in grey, and 84 in the yellow module. The cluster tree and the relationships between the modules are shown in Figure 5C,D. Figure 5E shows the connectivity in each module. The blue module was selected on account of its high correlation with tumor grade, but it had little to do with the gender and age of the patients (Figure 5F). 

### 3.4. Establish of Prognostic Model

**Figure 6:**
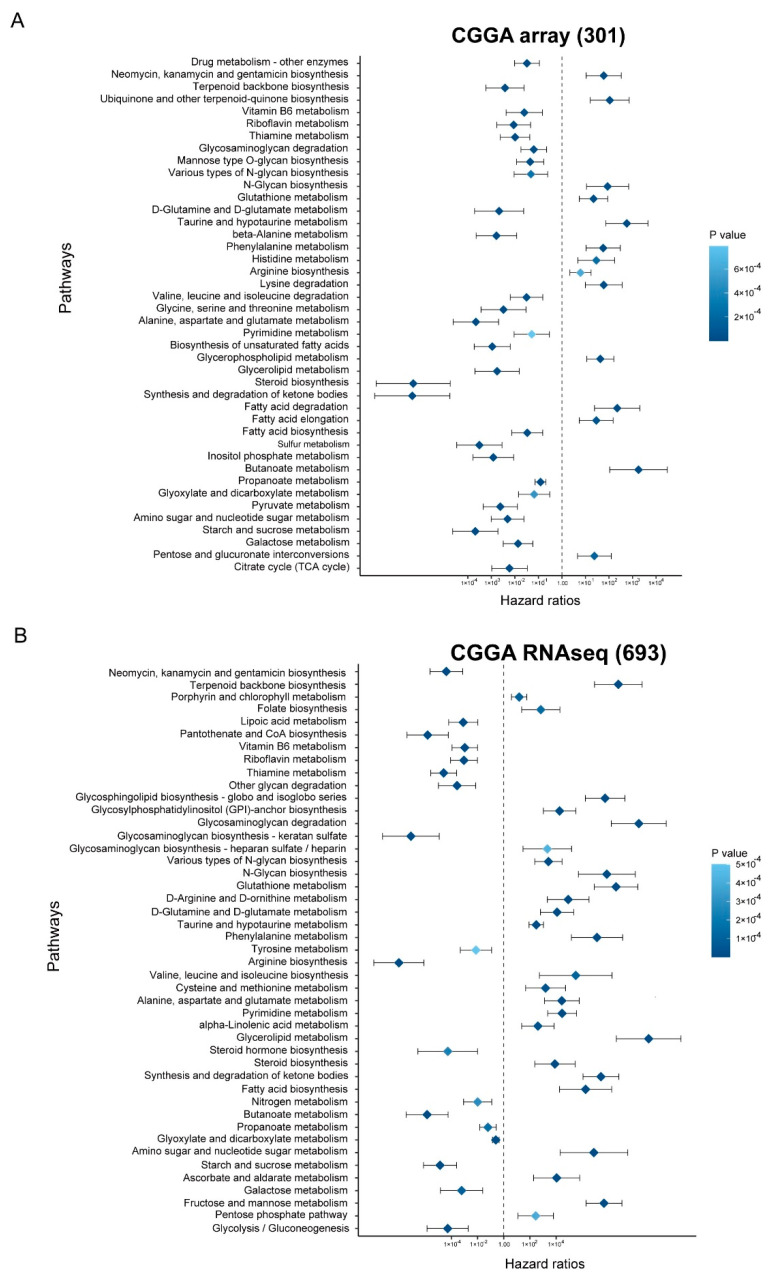
Metabolic activity associated with prognosis. (**A**,**B**) Hazard ratios of enrichment score in metabolic pathways with *p* < 0.0001 based on univariate Cox regression analysis in in CGGA array (301) and CGGA RNAseq (693) cohorts.

**Figure 7:**
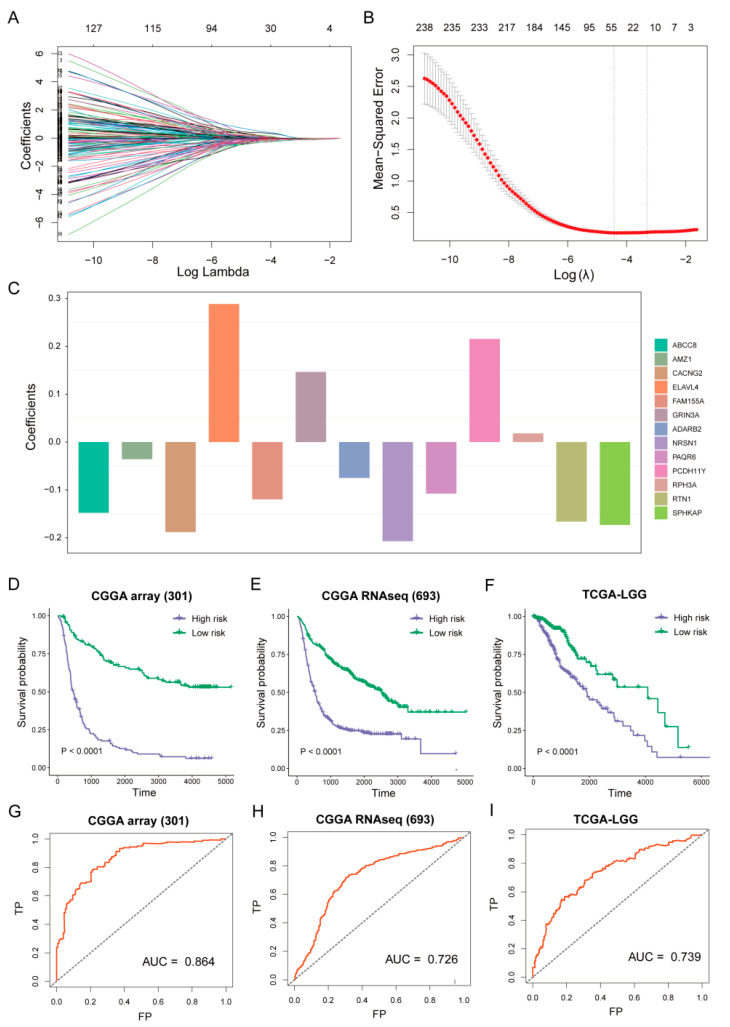
Construction of Metabolic prognostic model. (**A**) LASSO coefficient profiles of 239 prognostic genes. (**B**) Cross-validation of the LASSO model’s parameters. (**C**) The coefficients of 13 prognostic genes in predictive model. (**D**–**F**) In CGGA and TCGA cohorts, we used log-rank tests to determine whether high-risk and low-risk samples had different OSs. CGGA array (301): HR = 0.2004963; CGGA RNAseq (693): HR = 0.37367; TCGA-LGG: HR = 0.4145335. (**G**–**I**) ROC curve of prognostic model in CGGA and TCGA cohorts.

We evaluated the prognostic power of the metabolic pathway enrichment score, and the univariate Cox proportional hazard model revealed that metabolic activity was significantly relevant to prognosis in the CGGA and TCGA cohorts (Figure 6A,B and Figure S3, *p* < 0.001). To identify the genes that are capable of distinguishing patients with distinct prognoses, we conducted a univariate Cox proportional regression analysis for the blue module genes and found that 239 genes were statistically significantly correlated with OS. The most helpful prognostic genes were then determined using a LASSO analysis, and one SE above the minimal requirements was picked, resulting in a model with 13 prognostic genes (Figure 7A,B). Then, based on the expression of 13 genes, we established a predictive model according to a multivariate Cox proportional hazard model: risk score = (−0.2073 * NRSN1 exp) + (−0.1479 * ABCC8 exp) + (−0.1663 * RTN1 exp) + (0.07513 * ADARB2 exp) + (−0.1077 * PAQR6 exp) + (−0.1733 * SPHKAP exp) + (−0.1198 * FAM155A exp) + (0.1465 * GRIN3A exp) + (−0.1882 * CACNG2 exp) + (−0.03584 * AMZ1 exp) + (0.2154 * PCDH11Y exp) + (0.2885 * ELAVL4 exp) + (0.01816 * RPH3A exp) (Figure 7C).

A risk score was calculated for each patient in the CGGA and TCGA using the above formula. By using the median risk score as a cutoff value, the patients were categorized into high-risk and low-risk groups. There were significant differences in OS between the high-risk and low-risk groups in both the CGGA and TCGA studies (Figure 7D–F, *p* < 0.0001). According to the AUC (area under the curve) of the receiver operating characteristic (ROC) curve, we identified that the risk score was able to predict mortality accurately in the CGGA array (301) (AUC = 0.684), CGGA RNAseq (693) (AUC = 0.726), and TCGA-LGG (AUC = 0.739) (Figure 7G–I).

### 3.5. Risk Score Associated with Histological Subtypes

**Figure 8:**
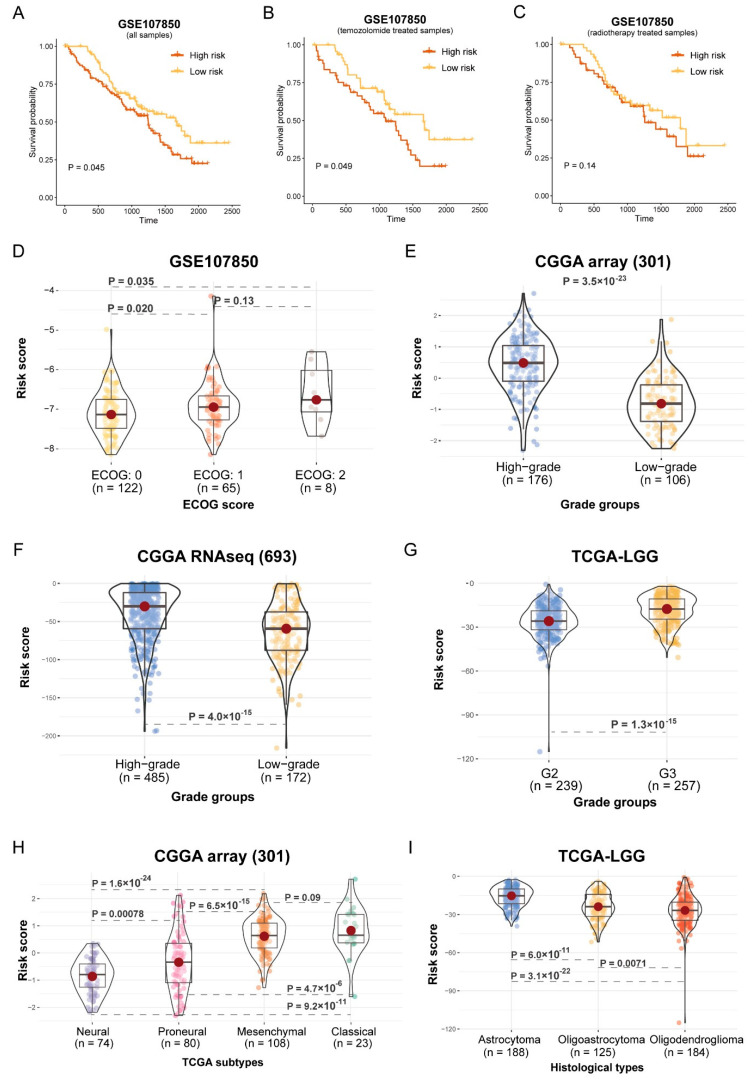
Risk score associated with clinical groups. (**A**–**C**) Assessment of the difference in OS between high-risk and low-risk samples in GSE107850 cohort within different therapy method (all patients, temozolomide-treated patients, and radiotherapy-treated patients) by log-rank test. (**A**): HR = 0.6703932; (**B**): HR = 0.5822906; (**C**): HR = 0.7964126. (**D**,**E**) The differences in risk scores within tumor grading groups and TCGA subtypes in CGGA array (301) cohort were assessed using the Wilcoxon rank-sum test. (**F**) The distribution of tumor gradings in the CGGA RNAseq cohort (693). (**G**) Comparison of risk score among ECOG score in GSE107850 cohort. (**H**,**I**) Differences in risk score between tumor grading groups and among histological types in TCGA-LGG cohort.

To confirm the power of the prognostic model under different treatment modalities, we compared the survival outcome of the patients in the high-risk and low-risk groups treated with chemotherapy and radiotherapy. The GSE107850 cohort had significantly poorer OS for all patients, as well as those treated with temozolomide (Figure 8A,B, *p* < 0.05), and the patients treated with radiotherapy had the same trend (Figure 8C, *p* = 0.14). The next step was to investigate the relationship between the risk score and therapeutic response. The ECOG performance status is a simple measure of the functional status of the patients, which contains three measures: score “0” means fully active and no performance restrictions; score “1” means strenuous physical activity restricted and fully ambulatory and able to carry out light work; score “2” means capable of all self-care but unable to carry out any work activities. We found that the risk score of the patients with an ECOG score of “2” were significantly greater than an ECOG score of “0” and an ECOG score of “1” in the GSE107850 cohort (Figure 8D, *p* < 0.05). The result revealed that the high-risk patients tend to have poorer outcomes and worse therapeutic responses. 

Moreover, we investigated the relationship between the risk score and histological subtypes of Glioma. The risk score significantly increased with high tumor grade in both the CGGA and TCGA cohorts (Figure 8E–G, *p* < 0.01). In addition, the risk scores of the four TCGA subtypes were significantly different in CGGA RNAseq (693) (Figure 8H, *p* < 0.05). The risk scores of the three histological types in the TCGA-LGG cohort were significantly different (Figure 8I, *p* < 0.05).

### 3.6. RPH3A Decreases LGG Cell Proliferation and Induce Apoptosis

**Figure 9:**
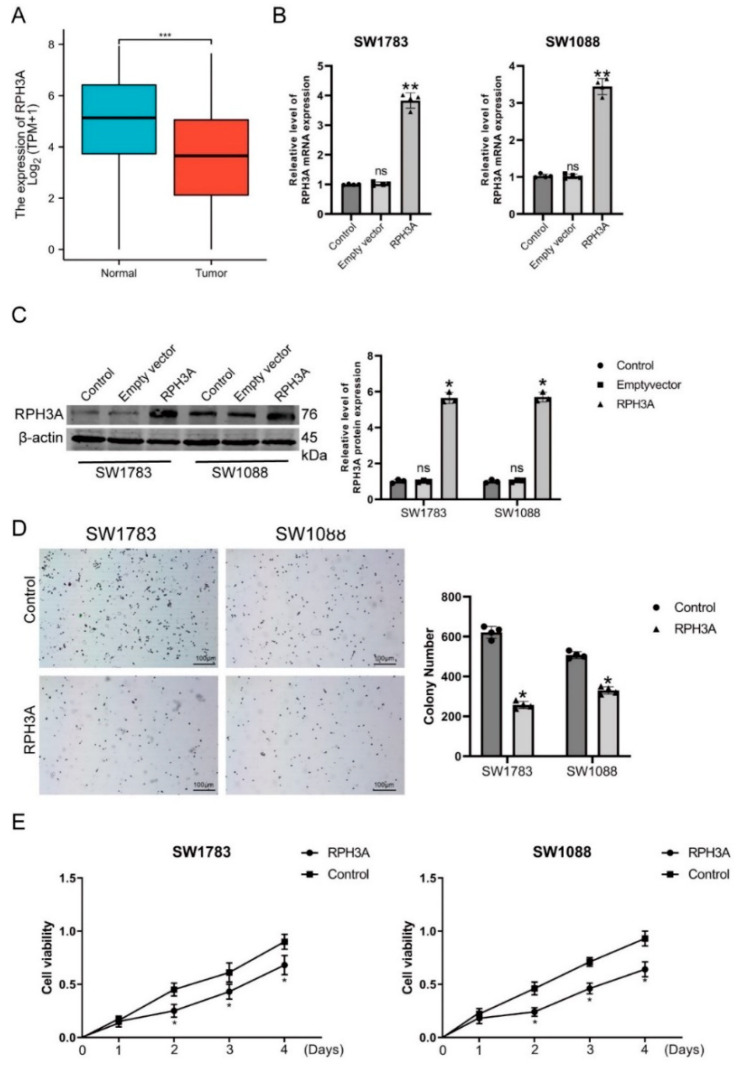
RPH3A alters the proliferation of LGG cells. (**A**). Relative expression of RPH3A in LGG and normal brain tissues. (**B**,**C**) Transfection efficiency of the RPH3A in SW1783 and SW1088 cells, measured by qRT-PCR and western blotting. (**D**) RPH3A-containing colonies showed reduced viability when compared to empty vector-containing colonies. (**E**) In CCK-8 assays, LGG cells expressing up-regulated RPH3A showed decreased cell viability. Means and standard deviations are represented as means and standard deviations. * *p* < 0.05, and ** *p* < 0.01 compare vs. Empty vector group, *** *p* < 0.001 compare vs. normal group. ns *p* > 0.05 compare vs control group.

Further validation of RPH3A’s involvement in tumor progression was performed using LGG. A comparison of the LGG samples with normal samples shows lower levels of RPH3A as expressed in the GEPIA database (~1.4) (Figure 9A and Figure S4). The expression of RPH3A in the SW1783 and SW1088 cells after transfecting with the RPH3A plasmid was tested using qRT-PCR and Western blotting. Our data show that, in LGG cells, RPH3A was significantly increased in the RPH3A overexpression group compared with the control group or empty vector group (Figure 9B,C). Consequently, the CCK-8 and colony formation assay were employed to test LGG cell growth and proliferation after treatment with RPH3A. A colony formation assay showed that the LGG cells, after overexpressing RPH3A, significantly decreased the colonies and colony volume (Figure 9D). Cell growth was evaluated by a CCK-8 assay after the overexpression of RPH3A in the LGG cells. Compared to the empty vector (NC) group, RPH3A overexpression can suppress SW1783 and SW1088 cell viability (Figure 9E). Thus, RPH3A affects cell growth and proliferation in LGG.

### 3.7. RPH3A Suppressed LGG Cell Migration, Invasion and EMT

**Figure 10:**
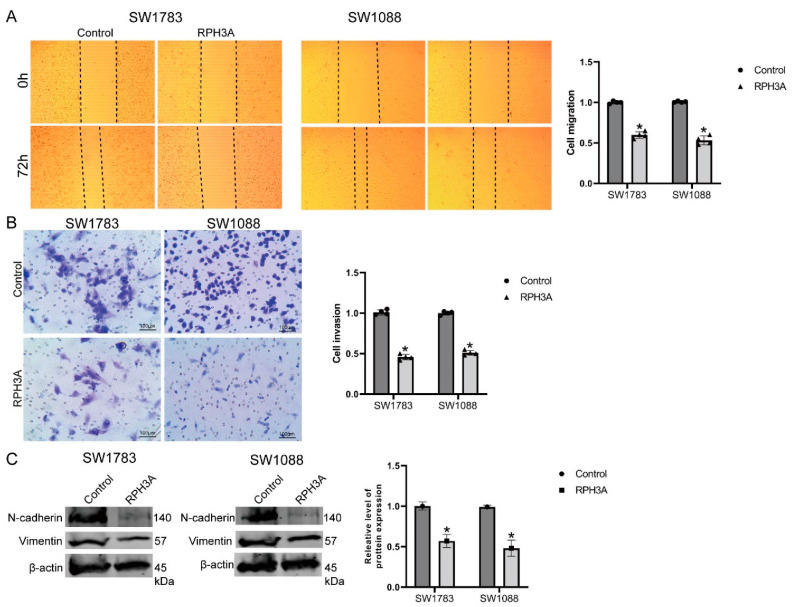
RPH3A suppressed migration, invasion, as well as EMT in LGG cells. After transfection with RPH3A in LGG cells. (**A**,**B**). Cell migration showed the signs of migration and invasion by wound healing assays as well as Trans well assays. (**C**). The N-cadherin and Vimentin protein levels in LGG cells after transfecting with RPH3A. In data analysis, mean values and standard deviations are calculated. * *p* < 0.05.

Wound healing and transwell migration assays were employed to test the role of RPH3A on migration and invasion in LGG. Our data showed the expression of RPH3A after increasing RPH3A in the LGG cells. Our data showed that invasive and migration activities were decreased in the LGG cells in the RPH3A group (Figure 10A,B). EMT-relative protein (N-cadherin and Vimentin) was detected in the LGG cells after the overexpression of RPH3A. Our data show that RPH3A overexpression can significantly decrease the protein level of N-cadherin and Vimentin in SW1783 and SW1088 cells compared to the NC group (Figure 10C). Consequently, RPH3A plays a role in LGG migration, invasion, and EMT.

## 4. Discussion

Although some studies have constructed some gene signatures [20,21,22,23,24], biomarkers for predicting the prognosis of LGG are still needed. The present study constructed a metabolic signature for Glioma classification. The data on Glioma were obtained from the CGGA, TCGA, GDC, and GEO databases. ssGSEA was utilized to calculate the enrichment score of each metabolic pathway. Based on the enrichment score, LGG was divided into two clusters. We then compared the outcomes between the two clusters. The results showed that cluster 1 has better OS than cluster 2 in the CGGA array and the CGGA RNAseq cohorts (Figure 1). The metabolic pathways were shown to have different activity between the two clusters (Figure 2). These results indicated that metabolic factors are related to the prognosis of LGG patients. 

We then observed the immune infiltration difference between the two clusters. The immune infiltration difference was observed between the two clusters. The 22 kinds of immune cells were analyzed using CIBERSORT. The infiltration of “T cells CD4 memory activated”, “T cells CD4 memory resting”, and “T cells follicular helper” in cluster 1 were significantly less than in cluster 2 in both the CGGA array and the CGGA RNAseq cohorts. While “Macrophages M0” and “B cells memory” in cluster 1 were significantly more than in cluster 2. It was reported that the number of CD4 T cells was larger than that in normal brain tissues. CD4 T cell infiltration-positive Glioma patients have better overall survival compared with CD4 T cell-infiltration negative [25]. Our results are in accordance with the current findings. 

The DEGs between the two subtypes were screened. A total of 688 DEGs were identified between the two subtypes. Pathway and process enrichment analyses were performed using these DEGs. The DEGs were enriched in some Glioma-related processes, including “regulation of ion transport”, “interferon-gamma signaling”, and “interleukin-4 and interleukin-13 signaling” (Figure 4). For example, the expression level of interferon-gamma was positively correlated with PD-L1 in Glioma. Moreover, interferon-gamma could enhance the expression of PD-L1, which may be an indicator for anti-PD-1/PD-L1 therapy [26]. Interleukin-4 is a Th2 cytokine that is related to the proliferation of lymphocytes [27]. Interleukin-4 could also promote tumor proliferation and aggressiveness [28,29]. The increased secretion of interleukin-4 was associated with macrophage-induced tumor growth and metastasis [30]. Polymorphisms in the interleukin-4 receptor genes are associated with better OS in Glioma patients [31]. The interleukin-4-related genes have a prognostic value for Glioma [32].

WGCNA was used to construct a co-expressed network using the DEGs, and four co-expressed modules were identified, which were the blue, green, grey, and yellow modules. The associations between these modules and clinical factors were analyzed. The blue module was highly correlated with tumor grade and had no correlation to the gender and age of the patients (Figure 5). 

We evaluated the prognostic power of the metabolic pathway enrichment score, and the univariate Cox proportional hazard model revealed that metabolic activity was significantly relevant to prognosis in the CGGA and TCGA cohorts (Figure 6). Subsequently, to identify the genes that could predict the prognosis of patients, we conducted a univariate Cox proportional regression analysis using genes in the blue module. A total of 239 genes were correlated with OS. Based on these genes, a prognostic model for LGG was established using LASSO Cox regression. A 13-gene-involved model was constructed, which included NRSN1, ABCC8, RTN1, ADARB2, PAQR6, SPHKAP, FAM155A, GRIN3A, CACNG2, AMZ1, PCDH11Y, and ELAVL4, RPH3A (Figure 7). Some of these genes are reported to be associated with the prognosis and progression of Glioma. NRSN1 was identified as a hub gene related to Glioma by Zhang et al. [33]. Zhou et al. found that ABCC8 mRNA expression could predict prognosis and chemosensitivity in Glioma [34].

According to the construct, the patients with Gliomas were classified into high-risk groups and low-risk groups. The patients in the high-risk groups had significantly worse OS in the CGGA and TCGA cohorts (Figure 7). Subsequently, we evaluated the role of the prognostic model in cancer treatment. Surgical resection, chemotherapy, and radiotherapy are the general treatment regimen for LGG. High recurrence and resistance rates are the main problem [35]. Our results showed that temozolomide- or radiotherapy-treated patients in the low-risk group had a better OS in the GSE107850 cohort. Next, we investigated the relationship between risk score and therapeutic response (Figure 8 A–C). We then evaluated the association between the risk score and the clinical pathological characteristics of the patients. The results showed that the risk score of the patients with an ECOG score of “2” were larger than those with an ECOG score of “0” and an ECOG score of “1” in the GSE107850 cohort (Figure 8D). The result revealed that high-risk patients tend to have poorer outcomes and worse therapeutic responses. Moreover, we investigated the relationship between risk score and histological subtypes of Glioma. The risk scores significantly increased with increasing tumor grade in the CGGA and TCGA cohorts (Figure 8E–G). In addition, the risk scores of four TCGA subtypes were different in CGGA RNAseq; the neural subtype had the lowest risk score, while the classical subtype had the highest risk score (Figure 8H). The risk scores of the three histological types in the TCGA-LGG cohort were also significantly different. The astrocytoma subtype has the highest risk score, while the Oligodendroglioma subtype has the lowest risk score (Figure 8I). This is consistent with the current clinical observations that the prognosis of Oligodendroglioma was better than astrocytoma [36]. Therefore, the risk score was associated with the clinical treatment and histological subtypes of LGG. 

Finally, the role of RPH3A in Glioma was detected. RPH3A is a functional gene that encodes rabphilin 3A and plays an essential role in neurotransmitter release and synaptic vesicle traffic. As the model component genes, RPH3A also plays an essential in immune microenvironment modulation. Ren C’s research indicated that RPH3A is an important regulator in the polarization of neutrophil [37]. RPH3A plays an important role in the process of neutrophil adhesion to endothelia during inflammation, which occurs through the regulation of RAB21 and PIP5K1C90 polarization [37,38]. The expression of RPH3A was increased in brain penumbra tissue of a rat cerebral ischemia-reperfusion model. The inhibition of RPH3A could aggravate brain injury. Therefore, the increase in RPH3A may be an endogenous protective mechanism against brain injury [39]. RPH3A was also shown to be a novel target for levodopa-induced dyskinesias [40]. However, the role of RPH3A in cancer remains unknown. We found that RPH3A was involved in our constructed prognostic model. RPH3A was decreased in LGG tissues. To further observe the effect of RPH3A on Glioma, RPH3A was overexpressed in SW1783 and SW1088 cells. The enhanced expression of RPH3A could inhibit the proliferation, migration, invasion, and EMT of LGG cells.

## 5. Conclusions

The present study developed a metabolic-related model for the prognosis of Glioma. This predictor may improve the prognosis of LGG. Additionally, RPH3A showed an anti-cancer effect on LGG.

## Data Availability

Data available on request due to restrictions i.e. privacy or ethical. The data presented in this study are available on request from the corresponding author.

## Supplementary Materials

The following supporting information can be downloaded at: https://www.mdpi.com/article/10.3390/brainsci12091138/s1, Figure S1: Stratification of LGG patients with distinct metabolic activity in TCGA; Figure S2: Divergenc of metabolic pathway activity and immune microenvironment between subtypes in TCGA-LGG cohort; Figure S3: Metabolic activity associated with prognosis in TCGA-LGG cohort. Figure S4: The expression of RPH3A in different types of organs; Table S1: Baseline characteristics of patients in CGGA and TCGA golima cohorts.
