# Supplementary material for: A Metabolic Plasticity-Based Signature for Molecular Classification and Prognosis of Lower-Grade Glioma
Source: Brain Sci. 2022 Aug 26;12(9):1138. doi: 10.3390/brainsci12091138 (PMC9497112; doi:10.3390/brainsci12091138)
Supplement: Supplementary file 1 [file brainsci-12-01138-s001.zip › brainsci-1790067-supplementary.pdf]

Supplementary Materials:

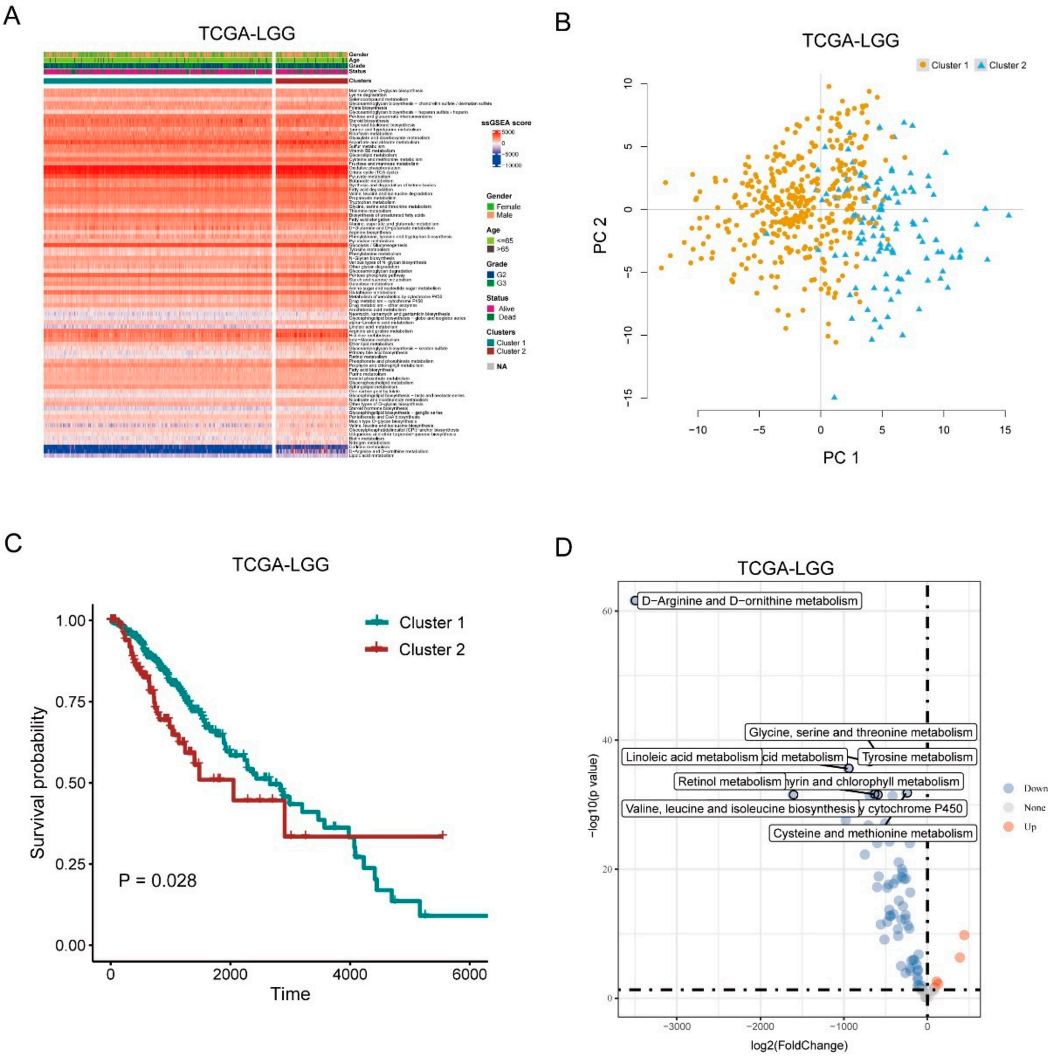

Figure S1. Stratification of LGG patients with distinct metabolic activity in TCGA.

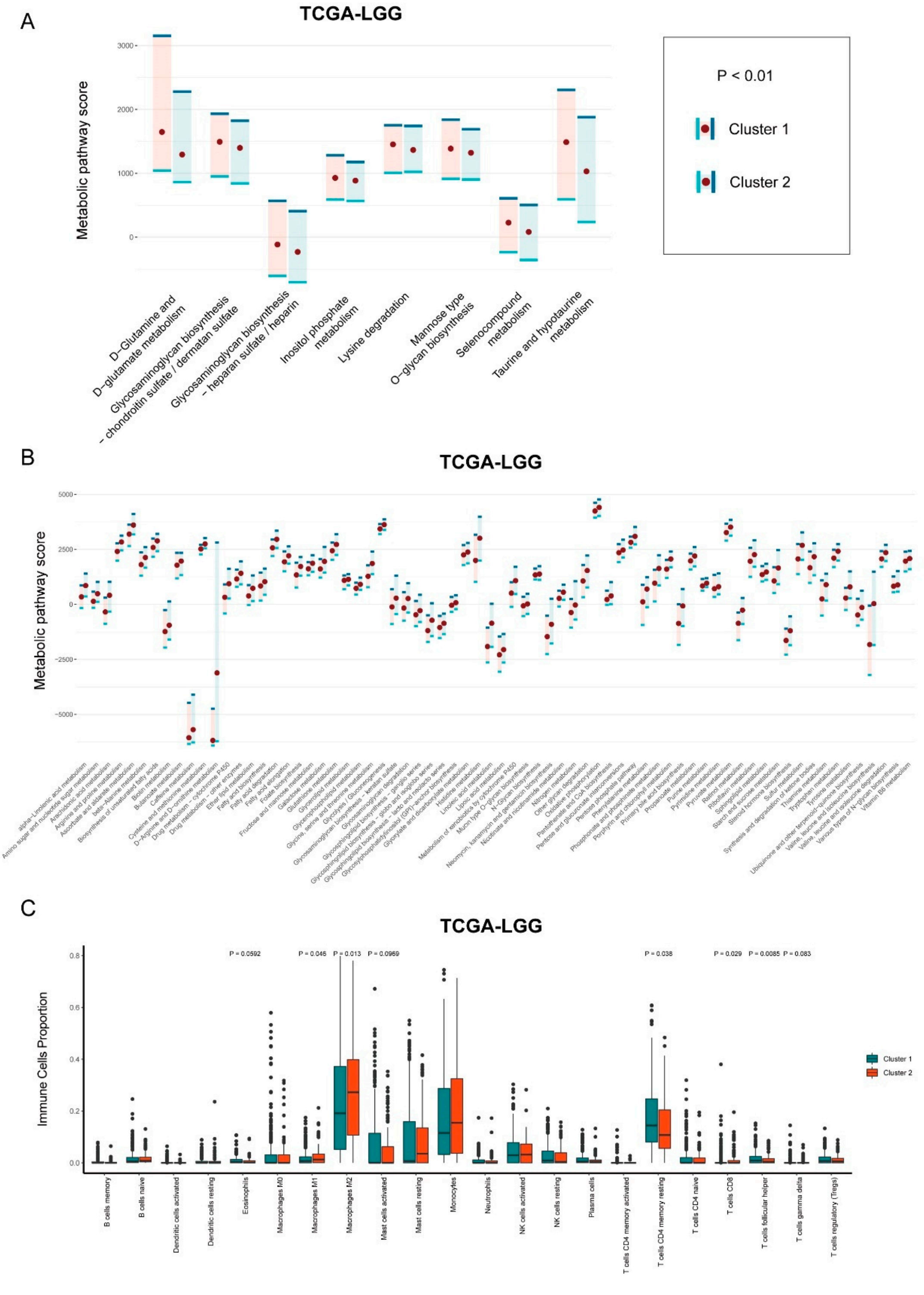

Figure S2. Divergenc of metabolic pathway activity and immune microenvironment between subtypes in TCGA-LGG cohort.

A

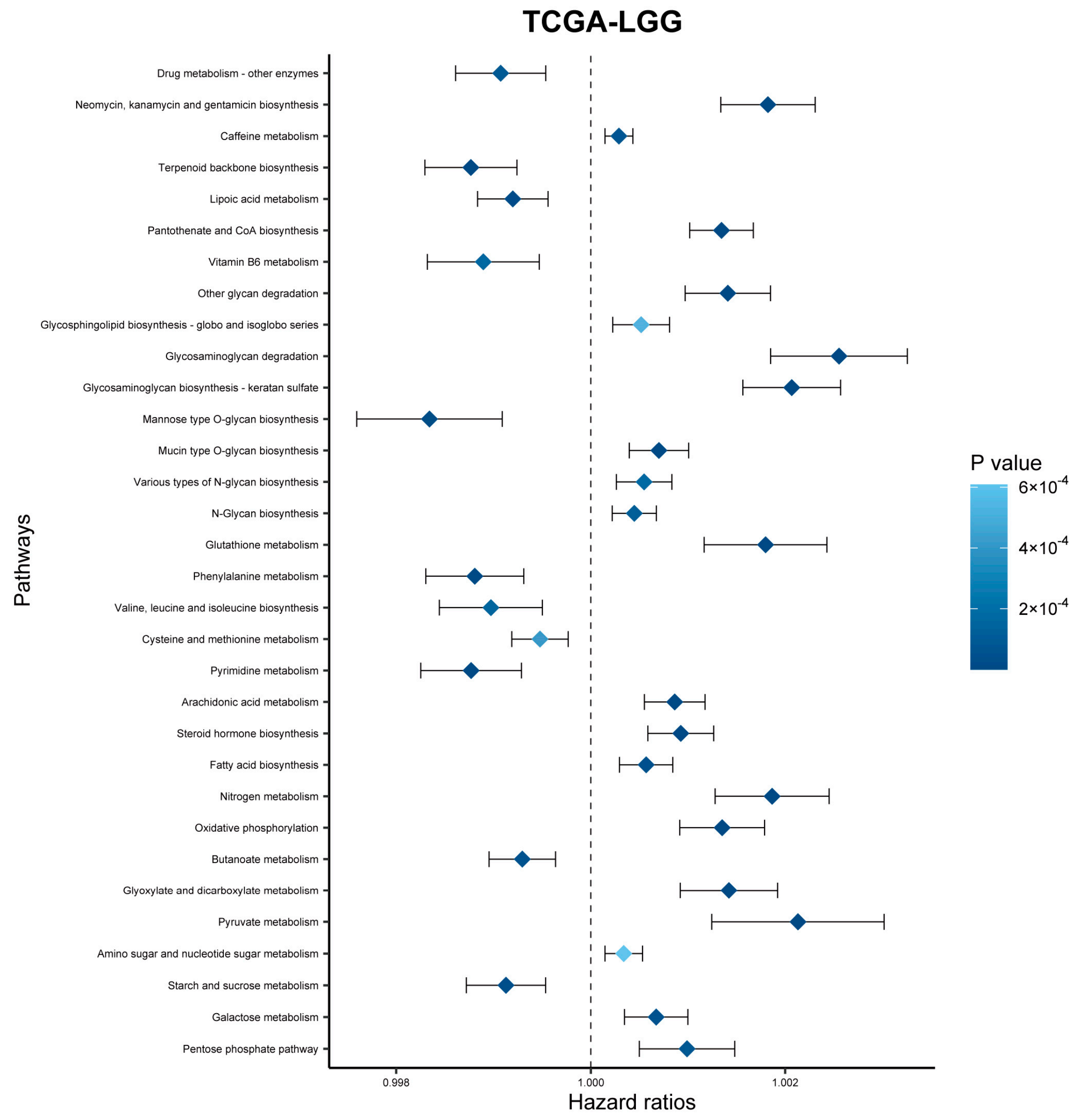

Figure S3. Metabolic activity associated with prognosis in TCGA-LGG cohort.

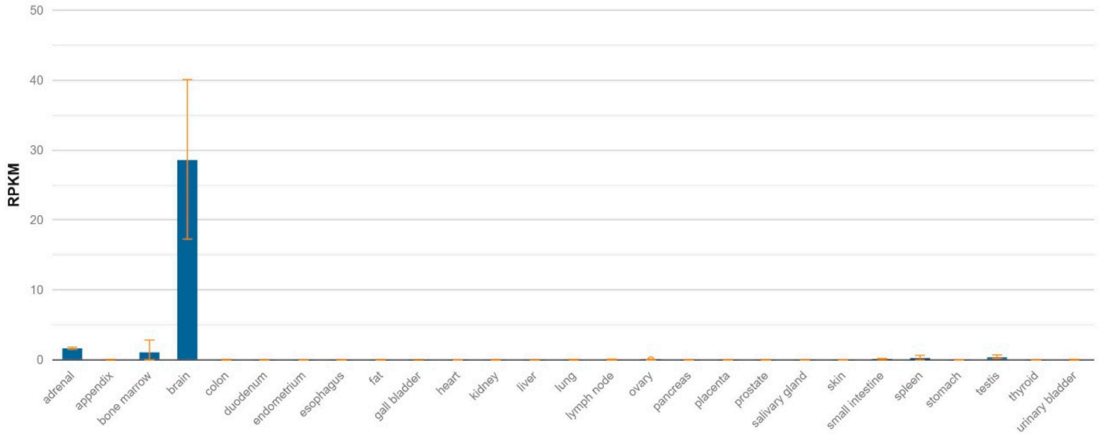

Figure S4. The expression of RPH3A in different types of organs.

Table S1. Baseline characteristics of patients in CGGA and TCGA golima cohorts.

Characteristics	Whole cohort	High risk	Low risk	*P*
**CGGA_array (301)**	(n = 285)	(n = 143)	(n = 142)	
**Gender**				0.064
Male	168(58.95%)	92(64.34%)	76(53.52%)	
Female	117(41.05%)	51(35.66%)	66(46.48%)	
**Age**				0.18
<65 years	278(97.54%)	138(96.5%)	140(98.59%)	
>=65 years	5(1.75%)	4(2.8%)	1(0.7%)	
**TCGA_subtypes**				$< 2.2\times10^{-16}$
Classical	23(8.07%)	20(13.99%)	3(2.11%)	
Mesenchymal	108(37.89%)	88(61.54%)	20(14.08%)	
Neural	74(25.96%)	6(4.2%)	68(47.89%)	
Proneural	80(28.07%)	29(20.28%)	51(35.92%)	
**Grade**				$< 2.2\times10^{-16}$
WHO II	106(37.19%)	16(11.19%)	90(63.38%)	
WHO III	53(18.6%)	24(16.78%)	29(20.42%)	
WHO IV	123(43.16%)	100(69.93%)	23(16.2%)	
**PRS_type**				0.093
Primary	250(87.72%)	121(84.62%)	129(90.85%)	
Recurrent	21(7.37%)	10(6.99%)	11(7.75%)	
Secondary	11(3.86%)	9(6.29%)	2(1.41%)	
**CGGA_RNAseq (693)**	(n = 657)	(n = 329)	(n = 328)	
**Gender**				0.88
Male	369(56.16%)	185(56.23%)	184(56.1%)	
Female	278(42.31%)	141(42.86%)	137(41.77%)	
**Age**				0.00061
<65 years	620(94.37%)	300(91.19%)	320(97.56%)	
>=65 years	36(5.48%)	28(8.51%)	8(2.44%)	
**Grade**				$< 2.2\times10^{-16}$
WHO II	172(26.18%)	46(13.98%)	126(38.41%)	
WHO III	248(37.75%)	106(32.22%)	142(43.29%)	
WHO IV	237(36.07%)	177(53.8%)	60(18.29%)	
**PRS_type**				2.8×10^{-10}
Primary	404(61.49%)	196(59.57%)	208(63.41%)	
Recurrent	253(38.51%)	133(40.43%)	120(36.59%)	
**TCGA-LGG**	(n = 497)	(n = 249)	(n = 248)	
**Gender**				0.90
Male	276(55.53%)	139(55.82%)	137(55.24%)	
Female	221(44.47%)	110(44.18%)	111(44.76%)	
**Age**				0.61
<65 years	462(92.96%)	230(92.37%)	232(93.55%)	
>=65 years	35(7.04%)	19(7.63%)	16(6.45%)	
**Grade**				4.5×10^{-12}
G2	239(48.09%)	81(32.53%)	158(63.71%)	
G3	257(51.71%)	167(67.07%)	90(36.29%)	
**Histological type**				$< 2.2\times10^{-16}$
Astrocytoma	188(37.83%)	141(56.63%)	47(18.95%)	
Oligoastrocytoma	125(25.15%)	50(20.08%)	75(30.24%)	
Oligodendroglioma	184(37.02%)	58(23.29%)	126(50.81%)	
